# Molecular cloning of ion channels in *Felis catus* that are related to periodic paralyses in man: a contribution to the understanding of the genetic susceptibility to feline neck ventroflexion and paralysis

**DOI:** 10.1242/bio.20148003

**Published:** 2014-07-25

**Authors:** Marlyn Zapata, Ilda S. Kunii, Rolf M. Paninka, Denise M. N. Simões, Víctor A. Castillo, Archivaldo Reche, Rui M. B. Maciel, Magnus R. Dias da Silva

**Affiliations:** Laboratory of Molecular and Translational Endocrinology, Division of Endocrinology, Department of Medicine, Escola Paulista de Medicina, Universidade Federal de São Paulo, São Paulo 04039032, SP, Brazil

**Keywords:** Potassium channel, Inward rectifier, *Felis catus*, Kir2.x, *KCNJ2*, *KCNJ12*, *KCNJ18*, *CACNA1S*, *SCN4A*, Cat

## Abstract

Neck ventroflexion in cats has different causes; however, the most common is the hypokalemia associated with flaccid paralysis secondary to chronic renal failure. In humans, the most common causes of acute flaccid paralysis are hypokalemia precipitated by thyrotoxicosis and familial forms linked to mutations in sodium, potassium, and calcium channel genes. Here, we describe the sequencing and analysis of skeletal muscle ion channels in *Felis catus* that could be related to periodic paralyses in humans, contributing to the understanding of the genetic susceptibility to feline neck ventroflexion and paralysis. We studied genomic DNA from eleven cats, including five animals that were hyperthyroid with hypokalemia, although only one presented with muscle weakness, and six healthy control domestic cats. We identified the ion channel ortholog genes *KCNJ2*, *KCNJ12*, *KCNJ14*, *CACNA1S* and *SCN4A* in the *Felis catus* genome, together with several polymorphic variants. Upon comparative alignment with other genomes, we found that *Felis catus* provides evidence for a high genomic conservation of ion channel sequences. Although we hypothesized that neck ventroflexion in cats could be associated with a thyrotoxic or familial periodic paralysis channel mutation, we did not identify any previously detected human channel mutation in the hyperthyroid cat presenting hypokalemia. However, based on the small number of affected cats in this study, we cannot yet rule out this molecular mechanism. Notwithstanding, hyperthyroidism should still be considered as a differential diagnosis in hypokalemic feline paralysis.

## INTRODUCTION

Ion channels are macromolecular protein complexes that are components of the cell membrane and are essentially important in different types of signaling, including transport, excitability, and conduction. Alterations in these channels may dynamically disturb cell and tissue physiology (Hille, 2001).

Indeed, there are many diseases related to ion channel dysfunction. In general, dominant and recessive mutations in genes encoding channels may lead to electrophysiological dysfunction, resulting in hyper- or hypo-excitability of the corresponding cells and typically causing so-called channelopathies (Abraham et al., 1999; Rolim et al., 2010; Jongsma and Wilders, 2001; Souto, 2011). Such altered genes can give rise to different clinical manifestations through gain-of-function (enhance) or loss-of-function (attenuate) mutations that affect the channel activity. Certain congenital disturbances affecting the skeletal muscle have been identified in humans and animals, such as disorders in calcium and potassium channels that can lead to paralysis and disorders in chloride channels that can lead myotonia; additionally, both paralysis and myotonia can originate from sodium and calcium channel disequilibrium.

Hypokalaemic Periodic Paralyses (HypokPP) include several uncommon diseases with clinical presentation characterized by acute and reversible attacks of muscle weakness, especially of the lower extremities, associated with low serum potassium. The most prevalent causes of HPP are Familial Hypokalaemic Periodic Paralysis (FHypokPP), an autosomal dominant disease, and an acquired form Thyrotoxic Hypokalaemic Periodic Paralysis (THypokPP), secondary to any cause of thyrotoxicosis. The symptoms of paralysis and the grade of hypokalaemia are almost identical in both FHypokPP and THypokPP, the differences in clinical features are related to the signs of thyrotoxicosis present in THPP (Rolim et al., 2010). Although work in human medicine has broadened the clinical spectrum by identifying several new channel mutations, such molecular genetic research on paralysis is seldom reported in veterinary medicine, and a promising feline animal model of THypokPP would be attractive for further genetic studies.

*Felis catus* is an interesting species for the study of human diseases. There are many instances in which the relationship between clinical signs, etiological agents, and molecular analysis of different pathologies has been established in cats (O'Brien et al., 1997a; O'Brien et al., 1997b), and the possible amino acid and gene sequence conservation among species throughout evolution may allow the identification of orthologous and syntenic genes that share a common evolutionary origin (Navratilova and Becker, 2009; Nomiyama et al., 2013; Ohno, 1973), therefore this animal model would shed light on the understanding of similar skeletal muscle diseases between man and cat.

Indeed, a comparison of genomes and analysis of synteny among ion channels using PCR, cloning, and sequence alignment would be useful for the molecular diagnosis of feline channelopathies. Here, we describe the channel genes *KCNJ2*, *KCNJ12*, *KCNJ14*, *SCN4A*, and *CACNA1S* in *Felis catus* in an attempt to associate the single-nucleotide polymorphisms (SNPs) found in these genes with feline ventroflexion and muscle paralysis.

## RESULTS AND DISCUSSION

We were able to study eleven cats including five hyperthyroid animals with hypokalemia, with only one presenting with muscle weakness, and six healthy control domestic cats, as summarized in Table 2. Since, familial hypokalemic periodic paralysis (FHypokPP) is an autosomal dominant disease associated with mutations in calcium channels *CACNA1S* (Cav1.1) and *SCN4A* (Nav1.4) (Kim et al., 2011; Sternberg et al., 1993), and sporadic/thyrotoxic hypokalemic paralysis is related to mutations in *KCNJ18* (Kir2.6) (Cheng et al., 2011; Maciel et al., 2011; Ryan et al., 2010; Silva et al., 2004; Wang et al., 2006), we approached principally these genes. The KCNJ18 mutants are primarily associated with THypokPP, an acquired genetic susceptibility condition in human.

**Table 1.**
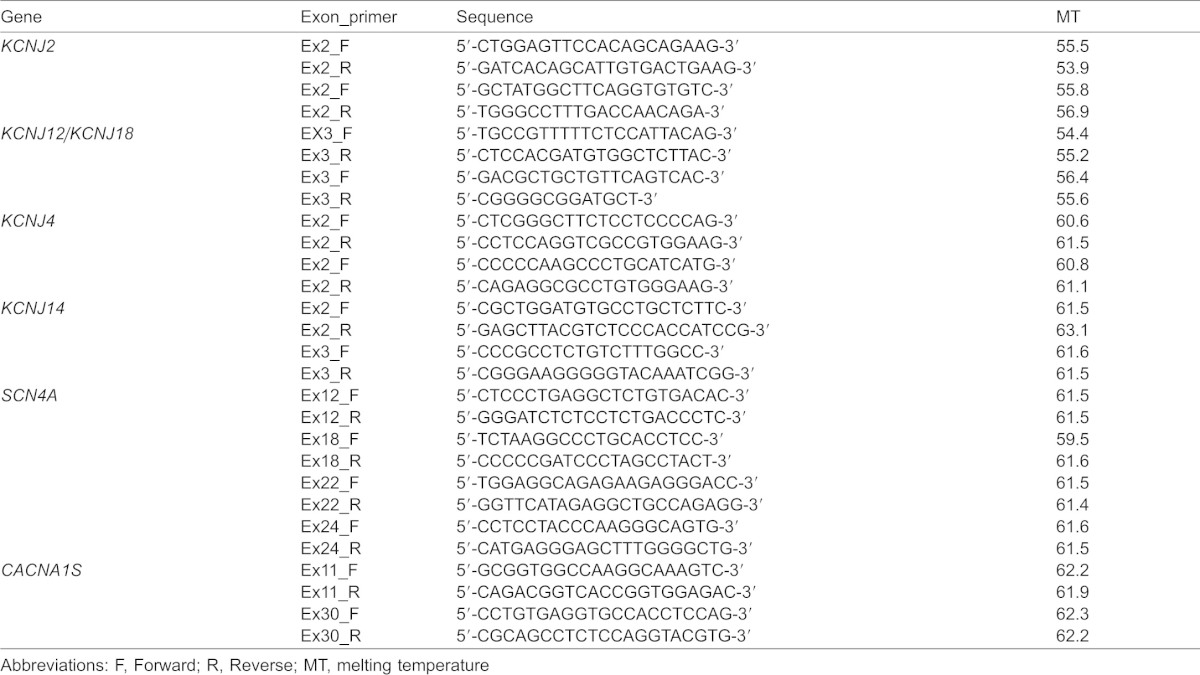
Details of primers used in this study for PCR, cloning and sequencing assays

**Table 2.**
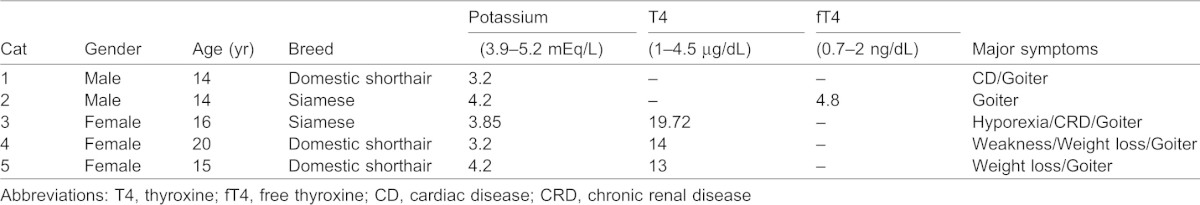
Epidemiological and physiopathological features of the studied cats diagnosed with goiter

### Molecular cloning of *fKir2.1*, *fKir2.2* and *fKir2.6* (or the human-like *KCNJ2*, *KCNJ12* and *KCNJ18* genes)

Of the neuromuscular disorders, cervical ventroflexion is a classic sign of generalized neuromuscular weakness in cats that can have different causes (Dickinson and LeCouteur, 2004). Cats, particularly the Burmese and Siamese breeds, exhibit paralysis associated with hypokalemia and muscle weakness similar to THypokPP in humans (Table 3) and has been termed hypokalemic periodic polymyopathy (HypoPP), sporadic feline hypokalemic polymyopathy, or periodic muscle weakness (Jones and Gruffydd-Jones, 1990; Lantinga et al., 1998). These conditions appear to be related to a sudden influx of potassium from the extracellular to intracellular compartment that is not accompanied by decreased potassium intake or increased renal potassium loss (Vite, 2002); however, the conditions have never been related to hyperthyroidism. Curiously, hypokalemia as a metabolic disease has also been detected in hyperthyroid cats (Crystal and Norsworthy, 2009; Dickinson and LeCouteur, 2004; Nemzek et al., 1994), although the potassium depletion in this state was generally considered secondary to reduced potassium ingestion or increases in the fractional excretion of potassium in urine (Fettman, 1989). Our study focusing on genes related to THypokPP in humans and our interest in identifying orthologous genes raises the hypothesis of *Felis catus* being the closest animal model for this skeletal muscle condition. Accordingly, we parallel THypokPP crisis in humans to ventroflexion plus paralysis in thyrotoxic hypokalemic cats.

**Table 3.**
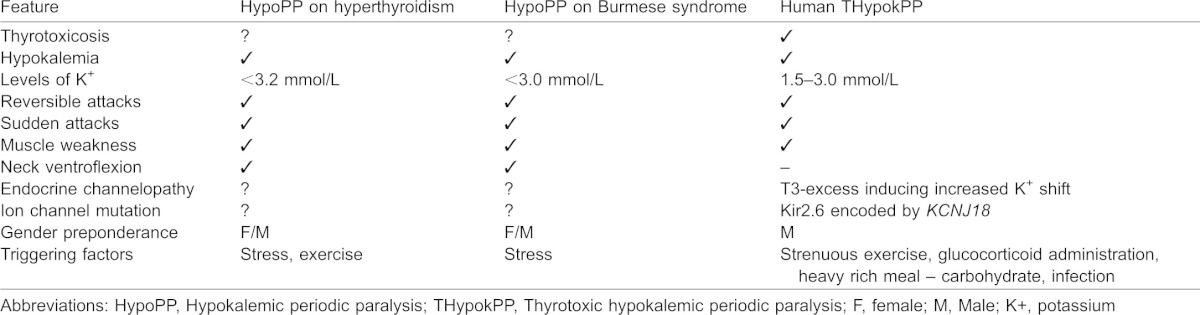
Major comparative features of hypokalemic periodic paralysis in felines and thyrotoxic periodic paralysis in humans

Based on the analysis of eleven domestic cats, we cloned the genes encoding Kir2.1 (*KCNJ2*), Kir2.2 (*KCNJ12*), and Kir2.6 (*KCNJ18*) from feline genomic DNA using human primers with low-stringency PCR and sequencing. We found that human Kir2.6 shares 96–99% amino acid identity with Kir2.2 and human Kir2.2 shares over 70% identity at the amino acid level with Kir2.1 (Ryan et al., 2010). We also found that the relationship between the two genes is preserved in cats and that the sequences are highly conserved between the species.

**Fig. 1.**
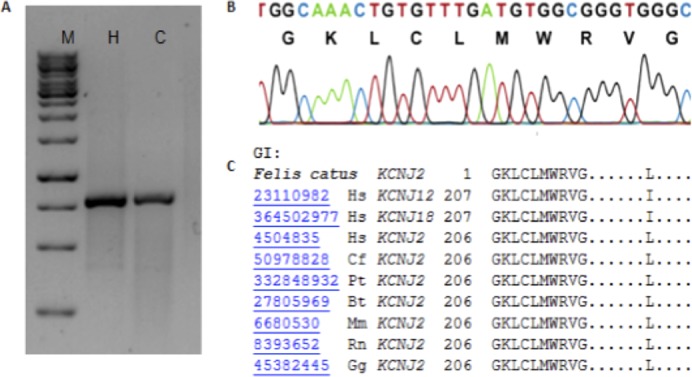
Identification of the *KCNJ2* (Kir2.1) gene in *Felis catus*. (A) Representative gel electrophoresis of the *KCNJ2* PCR product using the cat and human genomes. (B) Chromatogram of the region surrounding glycine 206 used for the comparative analysis (C) against other orthologous genomes available. Note that *f*KCNJ2 shows 91.1% identity with *Homo sapiens* (Hs), 90.6% with *Canis familiaris* (McFarland et al., 1980), 93% with *Pan troglodytes* (Pt), 91.6% with *Bos taurus* (Sternberg et al., 1993), 87% with *Mus musculus* (Prüss et al., 2005), 87.7% with *Rattus norvegicus* (Rn), and 84.6% with *Gallus gallus* (Ptáček et al., 1991). Abbreviations: (M) 1-kb molecular marker; cat (C) and human (H) PCR products.

**Fig. 2.**
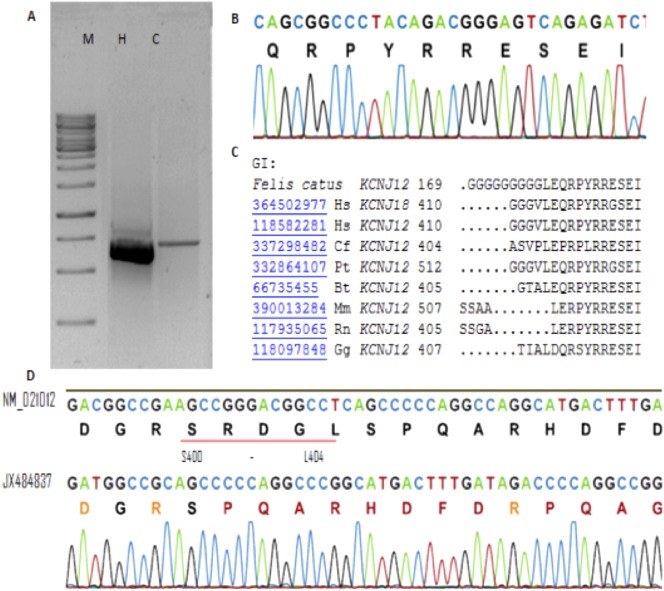
Identification of the *KCNJ12* (Kir2.2) gene in *Felis catus*. (A) Representative gel electrophoresis of the *KCNJ12* PCR product using the cat and human genomes. (B) Chromatogram of the region surrounding glutamine 424 used for comparative analysis (C) against other orthologous genomes available. Note that *fKCNJ12* shows 90% identity with *Homo sapiens* (Hs), 93% with *Canis familiaris* (McFarland et al., 1980), 90% with *Pan troglodytes* (Pt), 89% with *Bos taurus* (Sternberg et al., 1993), 86% with *Mus musculus* (Prüss et al., 2005), 85% with *Rattus norvegicus* (Rn), and 78% with *Gallus gallus* (Ptáček et al., 1991). (D) Five amino acids lacking in the cat sequence (red line); NM_021012 (human sequence) and JX484837 (cat sequence). Abbreviations: (M) 1-kb molecular marker; cat (C) and human (H) PCR products.

PCR amplification using primers designed for *hKir2.1* gene yielded a product of approximately 750 bp when examined on a 1% agarose gel (Fig. 1A). Its sequence represents the second exon of fKir2.1 (*fKCNJ2*) gene; this fragment included the feline coding region of the predicted human-like *KCNJ2* harboring 222 amino acids, which was then construed from the human sequence (GenBank: NG_008798). Primers designed based on the *hKir2.2* gene yielded a product of approximately 625 bp (Fig. 2A), which sequencing revealed that this fragment represents the third exon, comprising part of the coding region of fKir2.2 (*fKCNJ12*) gene, encoding 195 residues, which was also inferred to the human sequence (GenBank: NW_003315950.1).

Through the alignment of feline-like *KCNJ2* (750 bp) and *KCNJ12* (625 bp) segments in the NCBI *Felis catus* genome databank (http://blast.st-va.ncbi.nlm.nih.gov/Blast.cgi), we were able to uncover *in silico* the entire feline-like *KCNJ2* and *KCNJ12* genes (supplementary material Figs S1, S2, S3) in the *Felis catus* contig (GenBank: AANG02076766.1 and AANG02055501.1, respectively). Therefore, we designed specific primers corresponding to ends of both sequences to clone the full-length segments.

In addition, we compared *fKCNJ2* (Kir2.1) with other orthologues available in the NCBI database, revealing high conservation (Fig. 1C, Fig. 2C) among the cat Kir2.x family genes and among species. With regard to *fKCNJ12*, we observed five amino acids missing in comparison to human *KCNJ12* (Fig. 2D), S400–L404 in human Kir2.2; however, it is unclear how this loss would affect the channel in cats. Different polymorphisms were also found among different cats (Table 4). Although we attempted many strategies to identify the *KCNJ18* orthologous gene in the feline genome, we were unable to do so using DNA from peripheral blood lymphocytes or *in silico* prediction.

**Table 4.**
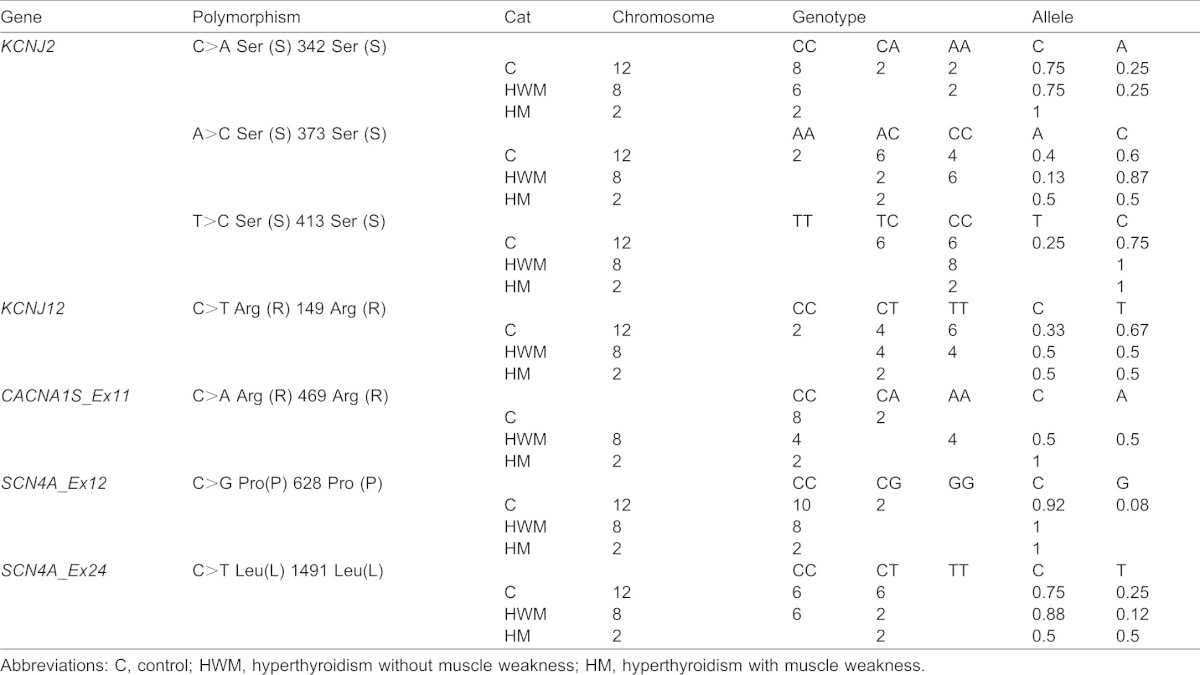
Genotype distribution of polymorphic variants found in *KCNJ2*, *KCNJ12*, *SCN4A* and *CACN1AS* in the group of hyperthyroid cats and control population

**Fig. 3.**
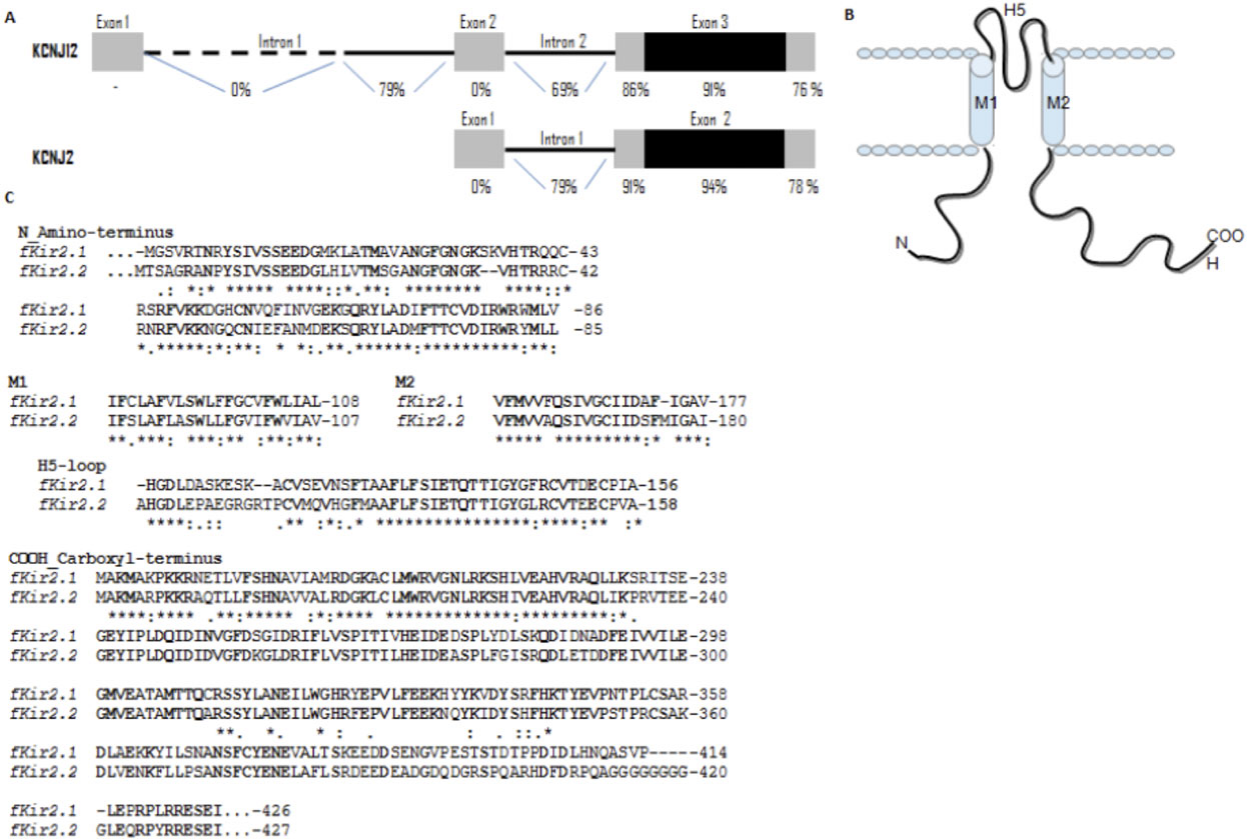
Schematic representation of the protein sequence alignment between *Felis catus* Kir2.1 and Kir2.2. (A) Comparative among human and *fKCNJ12* and *fKCNJ2* genes. We can observe 91% and 94% of homology in coding region among species. (B) General scheme of the Kir channel subunits. (C) Putative sequences shared are in bold, showing conserved sequence between subfamily members.

Inwardly rectifying K^+^ (Kir) currents were first identified in skeletal muscle (Hibino et al., 2010; Katz, 1949). Regarded as essential components for establishing a stable and negative resting membrane potential in many excitable cell types, these channel proteins (based on their subunits) act as inward current rectifiers, showing a greater flow of potassium into rather than out of the cell (Hugnot et al., 1997; Kaibara et al., 2002; de Boer et al., 2010); Kir channels regulate electrical excitability, repolarization of the action potential, and clearance of K^+^ from the T-tubule system (Kristensen et al., 2006). The functional subunits of Kir channels are tetrameric (i.e. four subunits), each of which has two membrane-spanning domains (M1 and M2), a flanking pore region (H5) (Hugnot et al., 1997), and cytoplasmic amino (NH_2_)- and carboxy (COOH)-terminal domains (Fig. 3B) (Hugnot et al., 1997; Kaibara et al., 2002).

The Kir channel family is encoded by the *KCNJ* genes (Dhamoon and Jalife, 2005) and includes seven subfamilies (Kir1-7) sharing 40–60% homology within each (de Boer et al., 2010). Different genetic diseases have been recognized in these channels: Andersen–Tawil syndrome caused by mutations in Kir2.1 (encoded by *KCNJ2*) (Obeyesekere et al., 2011; Plaster et al., 2001); cardiac cell, neuron, and muscle diseases caused by mutations in Kir2.2 (encoded by *KCNJ12*) (Hugnot et al., 1997; Prüss et al., 2005; de Boer et al., 2010); and susceptibility to thyrotoxic hypokalemic periodic paralysis (THypokPP) (Ryan et al., 2010), a clinical condition characterized by reversible attacks of muscle weakness associated with thyrotoxicosis, hypokalemia, and hypophosphatemia, caused by mutations in Kir2.6 (T354M, K366R, Q407X, and R399X) (encoded by *KCNJ18*) (Dias Da Silva et al., 2002; Maciel et al., 2011; Rolim et al., 2010; Ryan et al., 2010).

### Sequencing of hotspot regions for mutations causing human paralysis in the *Felis catus* genome: the *SCN4A* and *CACNA1S* genes

Familial hyperkalemic periodic paralysis (HYPP) is an autosomal dominant channelopathy generated by mutations in sodium channel *SCN4A* (Nav1.4) (Han and Kim, 2011; Ptáček et al., 1991), similar to HYPP in horses (Aleman, 2008). Other clinical manifestations of skeletal muscle identified to date are potassium-aggravated myotonia (PAM), paramyotonia congenita (PMC), hypokalemic periodic paralysis/familial hypokalemia periodic paralysis (HypoPP/FPP), and a form of congenital myasthenic syndrome (CMS).

**Fig. 4.**
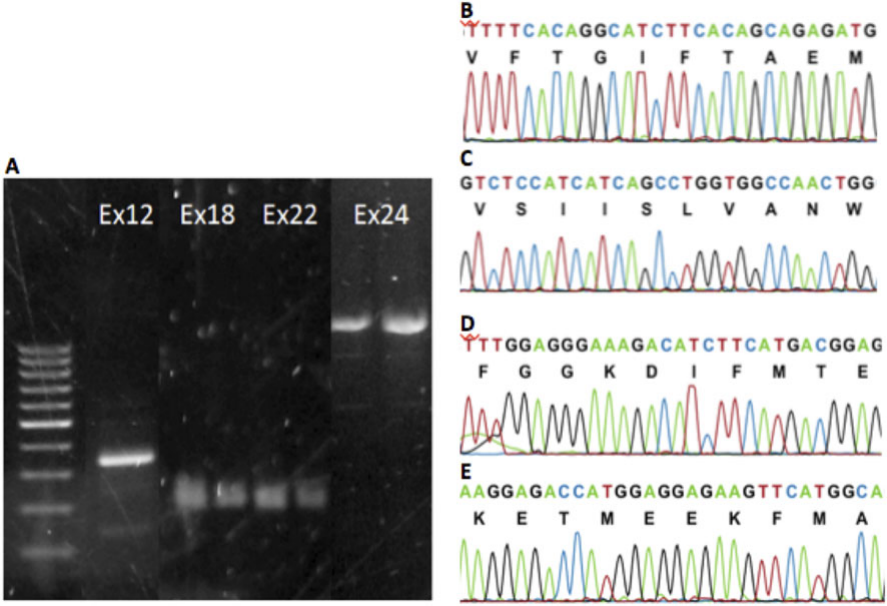
Identification of the *SCN4A* gene in *Felis catus*. (A) Representative gel electrophoresis of the *SCN4A* PCR product using the cat genome. We obtained products about for exon 12 (300 bp); exons 18 and 22 (200 bp); exon 24 (1.3 kb). (B–E) Chromatograms representative of exons 12, 18, 22, and 24, respectively. Comparison with other available orthologous genomes. Exon 12: *Homo sapiens* (Hs_97%), *Rattus norvegicus* (Rn_95%), *Mus musculus* (Mm_95%), *Gallus gallus* (Gg_98%), *Canis lupus familiaris* (Cf_97%), *Pan troglodytes* (Pn_97%), and *Bos taurus* (Bt_97%). Exon 18: 100% with *Homo sapiens*, *Rattus norvegicus*, *Mus musculus*, *Canis lupus familiaris*, and *Bos taurus*; *Gallus gallus* (Gg_93%); and *Pan troglodytes* (Pn_96%). Exon 22: *Homo sapiens* (Hs_97%); 100% with *Rattus norvegicus*, *Mus musculus*, *Gallus gallus*, *Canis lupus familiaris*, *Pan troglodytes*, and *Bos taurus*. Exon 24: *Homo sapiens* (Hs_94%); *Rattus norvegicus* (Rn_89%); *Mus musculus* (Mm_89%); *Gallus gallus* (Gg_85%); *Canis lupus familiaris* (Cf_98%); *Pan troglodytes* (Pn_94%); and *Bos taurus* (Bt_93%). Abbreviations: (Ex12) exon 12, (Ex18) exon 18, (Ex22) exon 22, and (Ex24) exon 24.

Using PCR with *in silico*-predicted feline primers, we were able to identify and confirm the *SCN4A* cat gene, specifically in relation to regions identified in association with several periodic paralysis mutations, including exons 12 (GenBank: KF267755), 18 (GenBank: KF267756), 22 (GenBank: KF267757), and 24 (GenBank: KF267758) (Fig. 4). Although no mutations were found with regard to divergent amino acid sequences compared to human, two polymorphisms were found in exons 12 and 24 (Table 4).

**Fig. 5.**
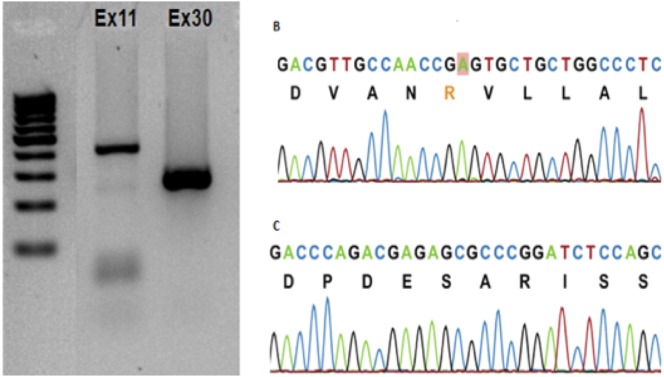
Identification of the *CACNA1S* gene in *Felis catus*. (A) Representative gel electrophoresis of the *CACNA1S* PCR product using the cat genome. (B,C) Chromatograms representative of exons 11 and 30, respectively. Comparison with other available orthologous genomes. Exon 11: *Homo sapiens* (Hs_86%); *Canis lupus familiaris* (Cf_92%); *Pan troglodytes* (Pn_88%); *Bos taurus* (Bt_92%); *Mus musculus* (Mm_88%); *Rattus norvegicus* (Rn_91%); and *Gallus gallus* (Gg_81%). Exon 30: 100% with *Homo sapiens*, *Canis lupus familiaris*, *Pan troglodytes*, and *Rattus norvegicus*; *Mus musculus* (Mm_95%); and *Gallus gallus* (Gg_81%).

By targeting hotspot regions in the human *CACNA1S* gene (GenBank: NM_001038605), we investigated the corresponding exons 11 and 30 in the cat genome, identifying polymorphisms in exon 11, as depicted in Table 4 and Fig. 5.

As both *CACNA1S* (Cav1.1) and *SCN4A* (Nav1.4) are related to HypoPP/FPP, autosomal dominant disorders causing either muscle weakness or flaccid paralysis with incomplete penetrance and occurring more frequently in young males, with later onset observed in affected females (Lin et al., 2005), we searched for mutations in our group of cats affected with muscle weakness. Although mutations typically affect segment S4 of domains II, III, and IV of *CACNA1S* and domains I, II, and III of *SCN4A* (Lin et al., 2010), no mutations in these genes (Table 5) were found in felines.

**Table 5.**
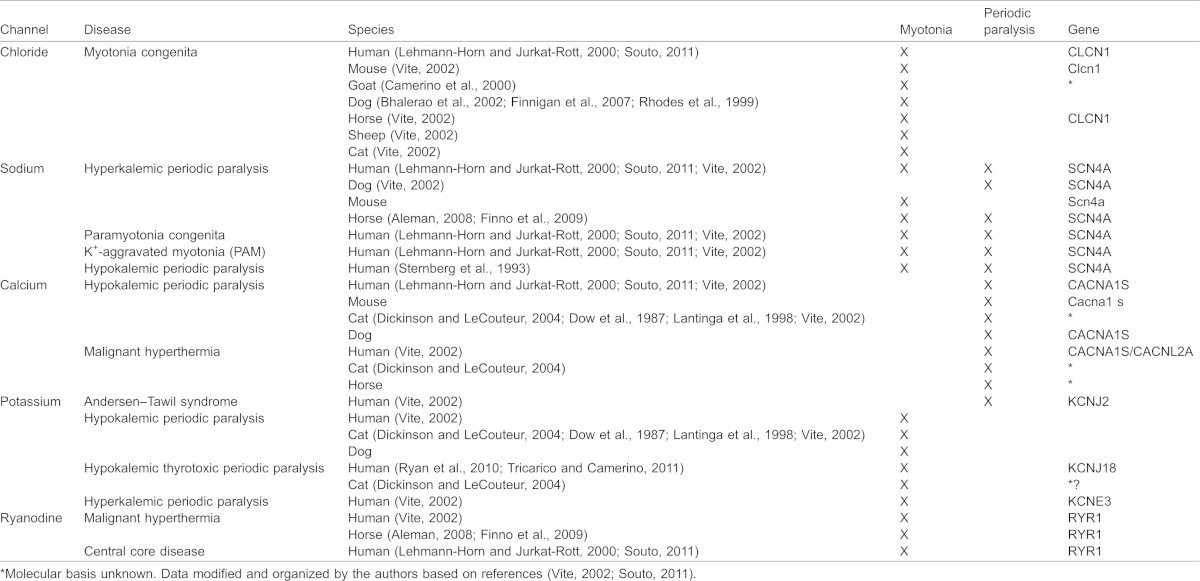
Comparative skeletal muscle channelopathies observed in mammals

### Channel genes *KCNJ2*, *KCNJ12*, *SCN4A* and *CACN1AS* appear to be in synteny

**Fig. 6.**
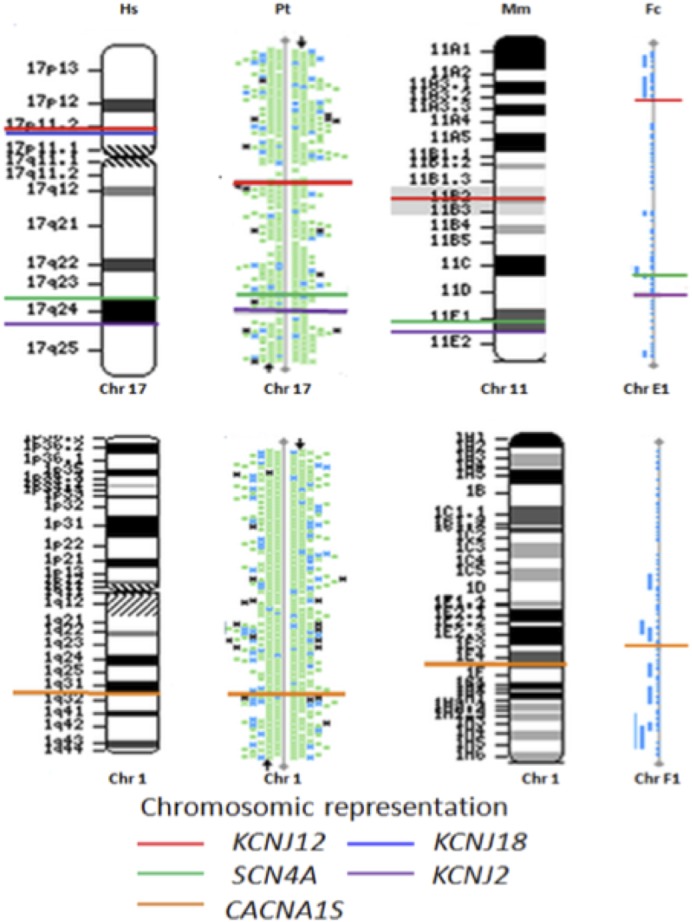
Channel genes *KCNJ12*, *KCNJ18*, *KCNJ2*, *SCN4A* and *CACN1AS* appear to be in synteny. Comparison between cat (*Felis catus*_Fc) genes with *Homo sapiens* (Hs), *Pan troglodytes* (Pt) and *Mus musculus* (Prüss et al., 2005). In humans, the *KCNJ12* and *KCNJ18* genes are located at chr17p11.2 (NM_021021.4, 21279699…21323179 and NM_001194958, 21308448…21320482, respectively). The *KCNJ12* gene: cat (chrE1, XM_003996356.1, 12629910…12638912); house mouse (chr11qB2, NM_001267593.1, 61022564…61073267); and chimpanzee (chr17, NC_006484.3; 34563412…34602842). The *KCNJ2* gene: human (chr17q24.3, NM_00891.2, 68165676…68176183), cat (chrE1, XM_003997110.1, 52625972…52633709), house mouse (chr11qE2, NM_008425.4, 11066164…11076825), and chimpanzee (chr17, XM_523701.4, 68959858…68967858). The *SCN4A* gene: human (chr17q23.3, NM_000334 -62015914…62050278), cat (chrE1, XM_003997064.1 -47941445…47968318), house mouse (chr11qE1, NM_133199.2, 106318593…106349390), and chimpanzee (chr17, XM_003315693.2, 62700479…62733665). The *CACNA1S* gene: human (chr1q32, NM_000069.2, 201008640…201081694), cat (chrF1q22, NM_001038605.1, 39372579…39437786), house mouse (chr1qE4, NM_014193.2, 136052901…136119822), and chimpanzee (chr1, XM_525018.3, 179792030…179866196).

Using NCBI tools (http://www.ncbi.nlm.nih.gov/projects/mapview), we were able to draw evolutionary genomic relationships (Fig. 6), as revealed by conservation in sequence identity (>85%), as colinearity by examining the chromosomal locations. The gene group comprising *KCNJ12*, *SCN4A*, and *KCNJ2* is located on chromosome 17 in humans and chimpanzees, chromosome 11 in mice, and chromosome E1 in cats; the *CACNA1S* gene is collinearly positioned on chromosome 1 in mice, chimpanzees, and humans and on chromosome F1 in cats. According to O'Brien et al., the syntenic block of feline chromosome E1 corresponds to human chromosome 17 and most likely murine chromosome 11 (O'Brien et al., 1997a); thereby it human 1 chromosome corresponds to C1 feline chromosome, which was reported by Pontius et al. (Pontius et al., 2007).

Our data reinforce the evolutionary structural genomic relationships among channel ortholog genes, which can suggest evolutionary gene duplication and further specialization of channel functioning. With respect to the *KCNJ18* gene (Kir2.6), we did not found this gene in either the genome of *Felis catus* or the *Mus musculus* C2C12 cell line; thus, we hypothesize that it might also be missing in the other lower mammal species. In fact, we were able to identify *in silico* Kir2.6 amino acid similarities in the predicted chimpanzee trace genome. Regardless, further studies need to be performed to confirm these findings because of the difficulty in distinguishing between Kir2.6 from Kir2.2 because they share high (98%) genomic homology, thereby limiting the ability to isolate them using ordinary sequencing methods.

Comparative genetic analysis has a high value for evolutionary, biological, and clinical aspects and allows the establishment of animal models for understanding interspecies diseases and explaining phenotype–genotype relationships. Although we did not discover any polymorphisms with clinical or causal relationships with paralysis/hypokalemia in the cat in this study, the DNA sequences of these genes are available for future studies. In the future, we hope to contribute more genetic studies on the diseases of *Homo sapiens*/*Felis catus*.

## MATERIALS AND METHODS

### Feline peripheral blood genomic DNA extraction

This study was conducted following the guidelines for Ethics Committee of the University Federal of São Paulo registered under CEP 1402/11. We enrolled 11 cats presented to Clinical Veterinary of Small Animal in São Paulo. DNA extraction was performed using a salting-out procedure according to an in-house method, as reported by Kizys et al. (Kizys et al., 2012).

### *Felis catus* PCR amplification using in silico predicted oligonucleotides

We initially used human primers previously designed for human potassium channel genes to pull out cat genomic sequence by using low-stringency PCR amplification since their sequence were unknown. The amplifications were performed using 100 ng feline genomic DNA in a 50-µl reaction containing 90% Platinum PCR SuperMix (Invitrogen, Carlsbad, CA). The reaction was as follows: an initial denaturation at 94°C for min, followed by 38 cycles of 94°C for 20 sec, 56°C annealing for 30 sec, and 72°C elongation for 1 min, and a final extension at 72°C for 5 min. For the PCR amplification of the feline *SCN4A* and *CACNA1S* genes, primers were designed *in silico* using NCBI human sequences, as shown in Table 1. These predicted primers were used in a 25-µl PCR reaction containing 100 ng/µl genomic DNA (1 µl) and 22.5 µl Platinum® PCR SuperMix (Invitrogen, Carlsbad, CA, USA); the reaction consisted of 35 cycles at 94°C for 5 min, followed by 94°C for 30 sec, 60°C for 45 sec, and a final extension at 72°C for 10 min. We applied different annealing temperatures for specific exons, as detailed in Table 1.

### Molecular cloning and sequence analysis of feline ion channels

The expected PCR bands were examined on a 1% agarose gel and then purified. The purified PCR product was cloned into pCR4®TOPO (Invitrogen Carlsbad, CA) and transformed into One Shot® MAX Efficiency® DH5α™-T1^R^ Competent Cells (Invitrogen, Carlsbad, CA). Positive clones were confirmed by sequencing using an ABI Prism 3100 Applied Biosystems Sequencer (CA, USA). The sequences were analyzed using BioEdit Sequence Alignment Editor and CLC Main Workbench 6 (http://www.clcbio.com).

### List of symbols and abbreviations used

PCR: polymerase chain reaction; SNPs: single-nucleotide polymorphisms; FHypokPP: familial hypokalemic periodic paralysis; ThyroKPP: thyrotoxic periodic paralysis; HypoPP: hypokalemic periodic polymyopathy; HYPP: familial hyperkalemic periodic paralysis; PAM: potassium-aggravated myotonia; PMC: paramyotonia congenital; HypoPP/FPP: hypokalemic periodic paralysis/familial hypokalemia periodic paralysis; CMS: congenital myasthenic syndrome; CEP: Ethics Committee of the University Federal of São Paulo; C: control; HWM: hyperthyroidism without muscle weakness; HM: hyperthyroidism with muscle weakness; CD: cardiac disease; CRD: chronic renal disease; F: forward; R: reverse; MT: melting temperature.

## Supplementary Material

Supplementary Material
