# Supplementary material for: Molecular cloning of ion channels in Felis catus that are related to periodic paralyses in man: a contribution to the understanding of the genetic susceptibility to feline neck ventroflexion and paralysis
Source: Biol Open. 2014 Jul 25;3(9):785–93. doi: 10.1242/bio.20148003 (PMC4163655; doi:10.1242/bio.20148003)
Supplement: Supplementary Material [file supp_3_9_785__index.html]

Molecular cloning of ion channels in Felis catus that are related to periodic paralyses in man: a contribution to the understanding of the genetic susceptibility to feline neck ventroflexion and paralysis — Molecular cloning of ion channels in Felis catus that are related to periodic paralyses in man: a contribution to the understanding of the genetic susceptibility to feline neck ventroflexion and paralysis — Supplementary Material 

# Molecular cloning of ion channels in *Felis catus* that are related to periodic paralyses in man: a contribution to the understanding of the genetic susceptibility to feline neck ventroflexion and paralysis

## bio.20148003 Supplementary Material

**Files in this Data Supplement:**

- Supplementary Material - Marlyn Zapata et al. doi: 10.1242/bio.20148003
