# Supplementary material for: Molecular cloning of ion channels in Felis catus that are related to periodic paralyses in man: a contribution to the understanding of the genetic susceptibility to feline neck ventroflexion and paralysis
Source: Biol Open. 2014 Jul 25;3(9):785–93. doi: 10.1242/bio.20148003 (PMC4163655; doi:10.1242/bio.20148003)
Supplement: Supplementary Material [file supp_bio.20148003_bio.20148003-s1.pdf]

Biology Open (2014) 000, 1–9 doi:10.1242/bio.20148003

Supplementary Material

Marlyn Zapata et al. doi: 10.1242/bio.20148003

```
MGSVRTNRYS IVSSEEDGMK LATMAVANGF GNGKSKVHTR QQCRSRFVKK DGHCNVQFIN
VGEKGQRYLA DIFTTCVDIR WRWMLVIFCL AFVLSWLFFG CVFWLIALLH GDLDASKESK
ACVSEVNSFT AAFLFSIETQ TTIGYGFRCV TDECPIAVFM VVFQSIVGCI IDAFIIGAVM
AKMAKPKKRN ETLVFSHNAV IAMRDGKACL MWRVGNLRKS HLVEAHVRAQ LLKSRITSEG
EYIPLDQIDI NVGFDSGIDR IFLVSPITIV HEIDEDSPLY DLSKQDIDNA DFEIVVILEG
MVEATAMTTQ CRSSYLANEI LWGHRYEPVL FEEKHYYKVD YSRFHKTYEV PNTPLCSARD
LAEKKYILSN ANSFCYENEV ALTSKEEDDS ENGVPESTST DTPPDIDLHN QASVPLEPRP
LRRESEI
```

```
atgggcagtgtgcgaaccaaccgctacagcattgtctcttcagaagaagacgggatgaag
 M  G  S  V  R  T  N  R  Y  S  I  V  S  S  E  E  D  G  M  K
ttggccaccatggcggttgcaaatggctttgggaatgggaagagtaaagtccacactcga
 L  A  T  M  A  V  A  N  G  F  G  N  G  K  S  K  V  H  T  R
caacagtgcaggagccgctttgtgaagaaagacggccactgtaatgttcagttcatcaat
 Q  Q  C  R  S  R  F  V  K  K  D  G  H  C  N  V  Q  F  I  N
gtgggtgagaagggacaacggtaccttgcagatatctttaccacatgtgtggacattcgc
 V  G  E  K  G  Q  R  Y  L  A  D  I  F  T  T  C  V  D  I  R
tggagatggatgctggttatcttctgtctggctttcgttctctcgtggctgtttttggc
 W  R  W  M  L  V  I  F  C  L  A  F  V  L  S  W  L  F  F  G
tgtgtgttttggttgatagctttgctccatggggatctggatgcatctaaagagagcaaa
 C  V  F  W  L  I  A  L  L  H  G  D  L  D  A  S  K  E  S  K
gcttgtgtgtcggaggtcaacagcttcacagctgccttcctcttctccatcgagacccag
 A  C  V  S  E  V  N  S  F  T  A  A  F  L  F  S  I  E  T  Q
acaaccatcggctatggcttcaggtgtgtcacggacgaatgcccaattgctgtttttatg
 T  T  I  G  Y  G  F  R  C  V  T  D  E  C  P  I  A  V  F  M
gtggtgttccagtccatcgtgggctgcatcattgatgcgtttatcattggcgcagtcatg
V  V  F  Q  S  I  V  G  C  I  I  D  A  F  I  I  G  A  V  M
gcaaagatggcaaagccaaagaagagaaacgagactcttgtcttcagtcacaatgctgtg
A  K  M  A  K  P  K  K  R  N  E  T  L  V  F  S  H  N  A  V
atcgccatgagagatggcaaagcgtgtttgatgtggcgggtgggcaaccttcggaaaagc
I  A  M  R  D  G  K  A  C  L  M  W  R  V  G  N  L  R  K  S
catttggtggaagctcacgtgagagcacagctgctcaaatccagaattacttccgaaggg
 H  L  V  E  A  H  V  R  A  Q  L  L  K  S  R  I  T  S  E  G
gagtacatcccctggatcaaatagatatcaacgttgggtttgacagtggaattgatcgc
 E  Y  I  P  L  D  Q  I  D  I  N  V  G  F  D  S  G  I  D  R
atatttctggtgtcccccatcactatagtccatgaaatagatgaagatagtcctttatat
 I  F  L  V  S  P  I  T  I  V  H  E  I  D  E  D  S  P  L  Y
gatttgagtaaacaggacattgacaatgcagactttgaaattgttgtgatactggaaggc
 D  L  S  K  Q  D  I  D  N  A  D  F  E  I  V  V  I  L  E  G
atggtggaagccacggccatgacaacacagtgccgtagctcatatctggccaatgaaatc
 M  V  E  A  T  A  M  T  T  Q  C  R  S  S  Y  L  A  N  E  I
ctttggggccaccgctatgagcctgtactctttgaagagaagcactattacaaagtggac
 L  W  G  H  R  Y  E  P  V  L  F  E  E  K  H  Y  Y  K  V  D
tactcaaggttccacaagacttatgaagtgcccaacactcccctttgtagtgccagagac
 Y  S  R  F  H  K  T  Y  E  V  P  N  T  P  L  C  S  A  R  D
ttagcagaaaagaaatatatcctctcaaatgctaattcattttgctatgaaaacgaagtt
 L  A  E  K  K  Y  I  L  S  N  A  N  S  F  C  Y  E  N  E  V
gccctcacaagcaaagaggaagatgacagtgaaaacggagtcccggaaagcactagtaca
 A  L  T  S  K  E  E  D  D  S  E  N  G  V  P  E  S  T  S  T
gacacacccccctgacatagaccttcacaaccaggcaagcgtacctctagagcccaggccc
 D  T  P  P  D  I  D  L  H  N  Q  A  S  V  P  L  E  P  R  P
ttacggcgagagtcggagatatgact
 L  R  R  E  S  E  I  -
```

Fig. S1. Full-length cDNA sequence of feline *KCNJ2* (Kir2.1). The amino acid sequence presented is that of the feline *KCNJ2* gene yielding a product of 427 aa. Coding sequence is capitalized with the corresponding amino acid above.

```
MTSAGRANPY SIVSSEEDGL HLVTMSGANG FGNGKVHTRR RCRNRFVKKN GQCNIEFANM
DEKSQRYLAD MFTTCVDIRW RYMLLIFSLA FLASWLLFGV IFWVIAVAHG DLEPAEGRGR
TPCVMQVHGF MAAFLFSIET QTTIGYGLRC VTEECPVAVF MVVAQSIVGC IIDSFMIGAI
MAKMARPKKR AQTLLFSHNA VVALRDGKLC LMWRVGNLRK SHIVEAHVRA QLIKPRVTEE
GEYIPLDQID IDVGFDKGLD RIFLVSPITI LHEIDEASPL FGISRQDLET DDFEIVVILE
GMVEATAMTT QARSSYLANE ILWGHRFEPV LFEEKNQYKI DYSHFHKTYE VPSTPRCSAK
DLVENKFLLP SANSFCYENE LAFLSRDEED EADGDQDGRS PQARHDFDRP QAGGGGGGGG
GLEQRPYRRE SEI
```

```
atgacctcggccggcagggccaacccctacagtatcgtgtcatcagaggaggacgggctg
 M  T  S  A  G  R  A  N  P  Y  S  I  V  S  S  E  E  D  G  L
cacctggtcaccatgtcgggcgccaacggcttcggcaacggcaaggtgcacacgcggcgc
 H  L  V  T  M  S  G  A  N  G  F  G  N  G  K  V  H  T  R  R
aggtgccgaaaccgcttcgtcaagaagaacggccagtgcaacattgagttcgccaacatg
 R  C  R  N  R  F  V  K  K  N  G  Q  C  N  I  E  F  A  N  M
gatgagaagtcgcagcgctacctggccgacatgttcaccacgtgcgtggacatccgctgg
 D  E  K  S  Q  R  Y  L  A  D  M  F  T  T  C  V  D  I  R  W
cgctacatgctgctcatcttctcgctggccttcctcgcctcctggctgctgttcggtgtc
 R  Y  M  L  L  I  F  S  L  A  F  L  A  S  W  L  L  F  G  V
atcttctgggtcatcgccgtggcccacggcgacctggagccggcagagggccgcggccgc
 I  F  W  V  I  A  V  A  H  G  D  L  E  P  A  E  G  R  G  R
acgccctgcgtgatgcaggtccacggcttcatggcggccttcctcttctccatcgagacg
 T  P  C  V  M  Q  V  H  G  F  M  A  A  F  L  F  S  I  E  T
cagaccaccatcggctacgggctgcgttgtgtgacggaggagtgccccgtggccgtcttc
 Q  T  T  I  G  Y  G  L  R  C  V  T  E  E  C  P  V  A  V  F
atggtggtggcgcagtccatcgtgggctgcatcatcgactccttcatgatcggcgccatc
M  VV  A  Q  S  I  V  G  C  I  I  D  S  F  M  I  G  A  I
atggccaagatggctcggcccaagaagcgggcacagacgctgctgttcagtcacaacgcc
M  A  K  M  A  R  P  K  K  R  A  Q  T  L  L  F  S  H  N  A
gtggtggccctgcgcgacggcaagctctgcctcatgtggcgcgtgggcaacctgcgtaag
V  V  A  L  R  D  G  K  L  C  L  M  W  R  V  G  N  L  R  K
agccacatcgtggaggcccacgtgcgggcccagctcatcaagccgagggtcaccgaggag
 S  H  I  V  E  A  H  V  R  A  Q  L  I  K  P  R  V  T  E  E
ggcgagtacatcccgctggaccagatcgacatcgatgtcggctttgacaagggcctcgac
 G  E  Y  I  P  L  D  Q  I  D  I  D  V  G  F  D  K  G  L  D
cgcatcttcctcgtgtcacccatcaccatcctgcatgagatcgacgaggccagcccgctg
 R  I  F  L  V  S  P  I  T  I  L  H  E  I  D  E  A  S  P  L
tttggcatcagccggcaggacctggagacagatgacttcgagatcgtggtcatcctggag
 F  G  I  S  R  Q  D  L  E  T  D  D  F  E  I  V  V  I  L  E
ggcatggtggaggccacggccatgaccacgcaggcccgcagctcctacctggccaacgag
 G  M  V  E  A  T  A  M  T  T  Q  A  R  S  S  Y  L  A  N  E
atcctgtggggccaccgctttgagcctgtcctctttgaggagaagaaccagtacaagatc
 I  L  W  G  H  R  F  E  P  V  L  F  E  E  K  N  Q  Y  K  I
gactactcccacttccacaagacctacgaggtgccctccacaccccgctgcagcgccaag
 D  Y  S  H  F  H  K  T  Y  E  V  P  S  T  P  R  C  S  A  K
gacctggtggagaacaaattcctgcttcccagtgccaactccttctgttacgagaacgag
 D  L  V  E  N  K  F  L  L  P  S  A  N  S  F  C  Y  E  N  E
ctggcctttctgagccgtgatgaggaggacgaggcagatggagaccaggatggccgcagc
 L  A  F  L  S  R  D  E  E  D  E  A  D  G  D  Q  D  G  R  S
ccccaggcccggcatgactttgatagaccccaggccggtggcggcggcggcggcggcggc
 P  Q  A  R  H  D  F  D  R  P  Q  A  G  GGGGGGG
ggcctcgagcagcggccctacagacgggagtcagagatctga
 G  L  E  Q  R  P  Y  R  R  E  S  E  I  -
```

Fig. S2. Full-length cDNA sequence of feline *KCNJ12* (Kir2.2). The amino acid sequence presented is that of the feline *KCNJ12* gene yielding a product of 433 aa. Coding sequence is capitalized with the corresponding amino acid above.

Biology Open (2014) 000, 1–9 doi:10.1242/bio.20148003

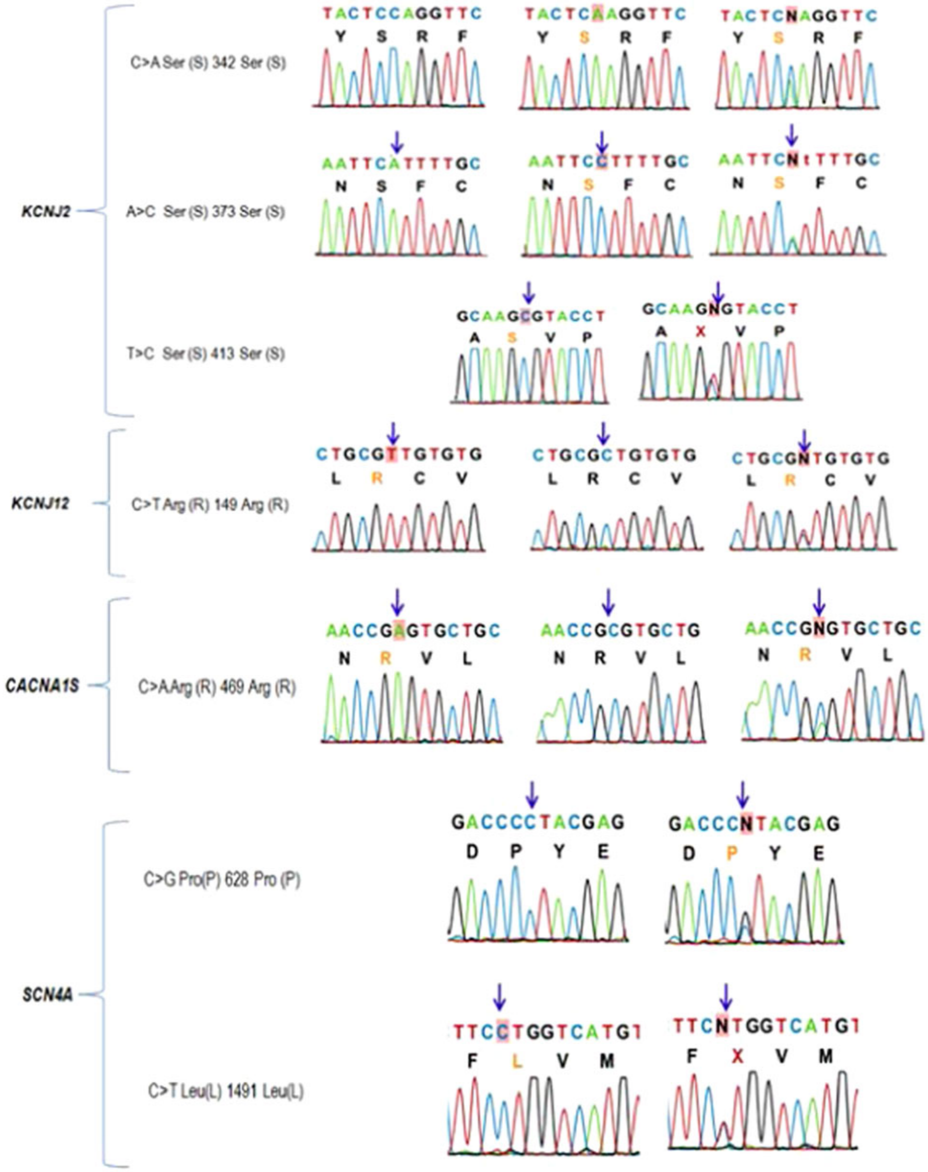

Fig. S3. Representative chromatograms of the polymorphisms (SNPs) identified in feline *KCNJ2*, *KCNJ12*, *SCN4A* and *CACNA1S* genes. All polymorphisms identified in hypokalemic hyperthyroid cats showed similar frequency in controls. Arrows indicate the polymorphic variants.
